# Identification of a linear B-cell epitope on the “puff” loop of the Senecavirus A VP2 protein involved in receptor binding

**DOI:** 10.3389/fmicb.2024.1387309

**Published:** 2024-04-23

**Authors:** Hanrong Zhou, Mingxia Sun, Shibo Su, Liang Meng, Wei Yang, Lan Yang, Xinqi Shi, Xin Li, Haiwei Wang, Hongwei Ma, Xuehui Cai, Yan-Dong Tang, Tongqing An, Fandan Meng

**Affiliations:** ^1^State Key Laboratory for Animal Disease Control and Prevention, Harbin Veterinary Research Institute of Chinese Academy of Agricultural Sciences, Harbin, Heilongjiang, China; ^2^College of Veterinary Medicine, Northeast Agricultural University, Harbin, China; ^3^Division of Nanobiomedicine, Suzhou Institute of Nano-Tech and Nano-Bionics, Chinese Academy of Sciences, Suzhou, China; ^4^Heilongjiang Research Center for Veterinary Biopharmaceutical Technology, Harbin Veterinary Research Institute, Chinese Academy of Agricultural Sciences, Harbin, China; ^5^Heilongjiang Provincial Key Laboratory of Veterinary Immunology, Harbin Veterinary Research Institute, Chinese Academy of Agricultural Sciences, Harbin, China

**Keywords:** Senecavirus A (SVA), B-cell epitopes (BCEs), monoclonal antibodies (mAbs), VP2 protein, receptor binding

## Abstract

Senecavirus A (SVA) is an important emerging swine pathogen that causes vesicular lesions in swine and acute death in newborn piglets. VP2 plays a significant role in the production of antibodies, which can be used in development of diagnostic tools and vaccines. Herein, the aim of the current study was to identify B-cell epitopes (BCEs) of SVA for generation of epitope-based SVA marker vaccine. Three monoclonal antibodies (mAbs), named 2E4, 1B8, and 2C7, against the SVA VP2 protein were obtained, and two novel linear BCEs, ^177^SLGTYYR^183^ and ^266^SPYFNGL^272^, were identified by peptide scanning. The epitope ^177^SLGTYYR^183^ was recognized by the mAb 1B8 and was fully exposed on the VP2 surface, and alanine scanning analysis revealed that it contained a high continuity of key amino acids. Importantly, we confirmed that ^177^SLGTYYR^183^ locates on “the puff” region within the VP2 EF loop, and contains three key amino acid residues involved in receptor binding. Moreover, a single mutation, Y182A, blocked the interaction of the mutant virus with the mAb 1B8, indicating that this mutation is the pivotal point for antibody recognition. In summary, the BCEs that identified in this study could be used to develop diagnostic tools and an epitope-based SVA marker vaccine.

## 1 Introduction

Senecavirus A (SVA), previously called Seneca Valley virus (SVV), is the only member of the genus *Senecavirus* in the family *Picornaviridae* (Hales et al., 2008; Zhang et al., 2018). SVA is a single-stranded positive-sense RNA virus associated with porcine idiopathic vesicular disease (PIVD) and epidemic transient neonatal loss (ETNL) (Vannucci et al., 2015; Leme et al., 2016); it has become an important swine pathogen and has caused significant economic losses. The clinical signs in SVA-infected pigs include ulcerative lesions on the snout and coronary bands, lameness, anorexia, lethargy, cutaneous hyperemia, and fever, affecting a high percentage of sows, finisher pigs, and neonatal piglets (Leme et al., 2017; Houston et al., 2020). The clinical manifestations and lesions of SVA are comparable to those of foot-and-mouth disease virus (FMDV), swine vesicular disease virus (SVDV), vesicular stomatitis virus (VSV), and vesicular exanthema of swine virus (VESV), which makes clinical diagnosis difficult. In 2015, SVA was first reported in China in Guangdong Province, and both adult and newborn pigs were infected during this outbreak (Wu et al., 2017). SVA began to spread rapidly among pigs in more regions and countries (Sun et al., 2018; Liu et al., 2020). In addition, co-infecting pigs with SVA and porcine reproductive and respiratory syndrome virus (PRRSV) may increase mortality (Paiva et al., 2023).

SVA is a non-enveloped RNA virus composed of an icosahedral capsid ~30 nm in diameter with a genome size of 7.2 kilobases (Hales et al., 2008). Like in other *picornaviruses*, SVA has one open reading frame (ORF) and encodes a single polyprotein precursor, which is then cleaved by protease into 12 functional proteins, including the lead protein L, structural protein P1 (VP1, VP2, VP3, VP4), nonstructural protein P2 (2A, 2B, 2C) and P3 (3A, 3B, 3C, 3D) (Houston et al., 2020). Among the SVA structural proteins, the VP1, VP2, and VP3 proteins are exposed on the virion surface and are responsible for stimulating host immune responses and binding to host receptors, while VP4 is located internally and interacts with internal nucleic acids (Strauss et al., 2018). In addition, VP2 is crucial for determining the cell tropism of SVA (Wang et al., 2023) and mediating virus binding to the cell receptor anthrax toxin receptor 1 (ANTXR1) (Miles et al., 2017). Although VP2 is the major structural protein that induces neutralizing antibodies (Dvorak et al., 2016; Maggioli et al., 2018; Wen et al., 2022), few neutralizing epitopes have been identified. Thus, identifying the epitopes of the SVA capsid protein VP2 has potential application value for analyzing the function and antigenicity of the VP2 protein.

In prior studies, several B-cell epitopes (BCEs) of SVA VP2 were identified by bioinformatics prediction (Ru et al., 2023; Zhang et al., 2023), overlapping synthesis of polypeptides (Ma et al., 2022) and monoclonal antibodies (mAbs) (Fan et al., 2020; Wen et al., 2022). It has been confirmed that ^153^QELNEE^158^ is a conserved antigenic epitope of the VP2 protein and has virus-neutralizing activity (Wen et al., 2022). The most common antigenic epitopes in the VP2 protein secondary structure are random coils, which include the EF loop (the “puff” structure) (Jayawardena et al., 2018), and the residues D166-G179, T180, and Y182-W187 in the SVA VP2 EF loop can recognize the cell receptor ANTXR1 (Miles et al., 2017; Cao et al., 2018). Neutralizing antibodies can block SVA infection, however, whether the receptor binding region on the VP2 EF loop contains the neutralizing BCEs of SVA has not been determined. In addition, Ma et al. (2022) identified highly conserved epitope peptides located at the C-terminus of VP2, the key amino acids not precisely defined in the present study.

In this study, we obtained three mAbs against the recombinant SVA VP2 protein and characterized the antigenic epitopes with a series of synthetic peptides and truncated proteins. The mAb 1B8 was bound at ^177^SLGTYYR^183^, and 2C7 was bound at ^266^SPYFNGL^272^ of VP2; the former is a novel epitope involved in the “puff” loop, while the latter corresponds to the carboxyl terminus of VP2. These two novel epitopes were identified for the first time by monoclonal antibodies. As vaccines currently remain the most effective methods for preventing SVA infection, characterization of SVA BCEs will provide novel insights into the development of diagnostic tools and the generation of a vaccine that enables the differentiation of infected individuals from vaccinated animals (DIVA).

## 2 Materials and methods

### 2.1 Cells, viruses, and plasmids

Baby hamster kidney fibroblast (BHK) 21 cells, the myeloma cell line SP2/0 and human embryonic kidney 293T cells were maintained in Dulbecco's modified Eagle's medium (DMEM; Gibco, USA) supplemented with 10% fetal bovine serum (FBS; Gibco, USA) at 37°C and 5% CO_2_ in a humidified atmosphere. The Senecavirus A strain (ZS) and the EGFP-expressing recombinant SVA used in this study were preserved at the Harbin Veterinary Research Institute (CAAS) (Jia et al., 2022). For SVA propagation, BHK-21 cells were infected with SVA at a multiplicity of infection (MOI) of 0.01 in DMEM and incubated at 37°C and 5% CO_2_ in a humidified atmosphere. When the cytopathic effect reached 80%, the cell supernatant was collected after being frozen and thawed 3 times. The SVA stocks were kept at −80°C. The pET-28a (+)-SUMO and pCAGGS-HA expression vectors were preserved in our laboratory. The full-length infectious cDNA clone pSVA16 was constructed previously (Li et al., 2019).

### 2.2 Expression and purification of the recombinant VP2 protein

Full-length SVA-VP2 was amplified, cloned and inserted into the pET-28a (+) expression vector to prepare purified SVA-VP2 proteins as an immunogen. For purification and protein solubility purposes, recombinant VP2 proteins were induced with 6 × His and SUMO tags. The pET28a-SUMO-VP2 recombinant plasmid was transformed into *E. coli* BL21 (DE3) competent cells. When the OD600 reached 0.6, the protein expression was induced by 0.1 mmol/L IPTG for 18 h at 16°C. The bacterial cells were harvested by centrifugation at 8,000 × g for 10 min and resuspended in PBS. Then, sonication was performed with an ultrasonic cell disruptor (Cole Parmer, Vernon Hills, IL, USA). Thereafter, the recombinant VP2 protein was analyzed via SDS–PAGE. The soluble recombinant VP2 protein was purified, primarily by nickel affinity chromatography and precisely by a size-exclusion chromatographic column in an automated system (AKTA, GE-Healthcare Life Sciences, USA). The concentration of purified protein was measured by a BCA protein concentration determination kit (Thermo Fisher Scientific, USA), and the proteins were stored at −80°C.

### 2.3 SVA purification

SVA particles were purified according to methods adapted from previously published protocols (Reddy et al., 2007; Jayawardena et al., 2018; Strauss et al., 2018). Briefly, SVA-infected BHK-21 cells were harvested for virus purification when a complete cytopathic effect was observed. After three freeze–thawing steps, the cell lysate was centrifuged at 8,000 rpm for 30 min at 4°C to remove cellular debris. Subsequently, the supernatant was transferred to 70 mL polypropylene centrifuge tubes (catalog no. 355622; Beckman Coulter) and centrifuged at 80,000 × g for 3 h in a Beckman Coulter SW 45 Ti rotor at 4°C. The virus pellet was resuspended in 1 ml of cesium chloride (CsCl) buffer (20 mM Tris-HCl, 1 mM EDTA, pH 7.8). For purification, the virus was isolated by CsCl gradient ultracentrifugation. The suspended virus was loaded on top of a CsCl gradient (concentration from top to bottom: 1 mL, 1.30 g/mL; 10 mL, 1.33 g/mL; and 1 mL, 1.40 g/mL) prepared in a 13.2 mL open-top polypropylene tube (catalog no. 331372; Beckman Coulter) and centrifuged at 61,580 × g for 18 h in a Beckman Coulter SW 41 Ti rotor at 22°C. The virus band was slowly collected for further identification via SDS–PAGE and TEM. The concentration was measured using spectrophotometry, and small aliquots of the virus were stored in sterile tubes at −80°C.

### 2.4 Generation of monoclonal antibodies

The animal experiments were approved by the Animal Care and Ethics Committees of Harbin Veterinary Research Institute, Chinese Academy of Agricultural Sciences. The methods were conducted in accordance with the approved animal ethics guidelines (number 210119-02). Six-week-old specific pathogen-free (SPF) female BALB/c mice were obtained from Beijing Weitong Lihua Laboratory Animal Technology. The purified recombinant VP2 protein was used as an immunogen emulsified with equal volumes of the adjuvant Montanide ISA-201 (SEPPIC, France). Mice were immunized with 50 μg of emulsified VP2 proteins three times on Days 0, 14, and 28 by intramuscular injection in the inner thigh. One week after the third vaccination, serum samples were collected, and antibody titers were detected via indirect ELISA. When the antibody titer reached more than 1:10,000, the mice were intraperitoneally injected with recombinant VP2 protein.

On the 3rd day after booster vaccination, the mice were humanely euthanized, and the spleen was aseptically extracted for spleen lymphocyte isolation. Splenocytes were fused with SP2/0 cells by 50% (w/v) PEG4000 (Sigma–Aldrich) according to the standard procedure (Galfrè and Milstein, 1981). The fused cells were cultured in medium containing HAT (Sigma–Aldrich) selection medium for 1 week, after which the medium was changed to HT medium until the 10th day. The supernatants of the hybridomas were screened by an indirect ELISA on a purified SVA precoated microplate. Positive hybridomas were subcloned at least three times by a limited dilution method to obtain a single hybridoma cell line. Ascites fluid was produced by intraperitoneal injection of positive hybridoma cells (10^5^-10^6^ cells/mouse) in liquid paraffin pre-treated BALB/c mice, and purified by Protein G affinity column (GenScript, Nanjing, China).

### 2.5 Enzyme-linked immunosorbent assay

Recombinant VP2 protein, purified virions and synthetic peptides were precoated onto microplates for indirect ELISA to determine the reactivity and epitopes of the monoclonal antibodies. Briefly, a 96-well ELISA plate was coated with 100 ng of the above antigen at 4°C overnight and then blocked with 5% (w/v) skim milk in PBS at 37°C for 2 h. A total of 100 μL of the hybridoma cell supernatant was added to each well and incubated at 37°C for 1 h. After washing with PBST, an HRP-conjugated goat anti-mouse IgG antibody (diluted 1:10,000 in PBST) was added to each well and incubated at 37°C for 1 h. The plates were washed with PBST before adding TMB Chromogen Solution (Abcam) as an enzyme substrate, and 2 M HCl was used to terminate the reaction. After color development, the optical density (OD) was measured by a microplate reader (Bio-Rad) at 450 nm. Furthermore, the antibody isotype was analyzed by the Antibody Isotyping Assay Kit (Proteintech, USA).

### 2.6 Indirect immunofluorescence assay

BHK-21 cells that reached 100% confluence were infected with EGFP-SVA at an MOI of 0.1 for 24 h. Then, the cells were washed with PBS, fixed with 3.7% paraformaldehyde for 20 min and permeabilized with 0.1% Triton X-100 for 20 min at room temperature. After blocking with 2% BSA for 1 h, the cells were incubated with 100 μL of hybridoma cell supernatant for 1 h. After washing three times with PBS, 100 μL of Alexa Fluor 568 dye-conjugated goat anti-mouse IgG (H + L) (red) at a dilution of 1:1,000 was added to each well for 1 h at room temperature. Finally, the cell nuclei were stained with 4′,6-diamino-2-phenylindole (DAPI; Sigma) after washing three times with PBST. The samples were then observed with a fluorescence microscope (EVOS F1, AMG, USA).

### 2.7 Western blot

The recombinant VP2 protein, vector protein, SVA and cell control were separated via 12% SDS–PAGE and subsequently transferred onto a PVDF membrane (Millipore, MA, USA). After blocking with 5% skim milk powder at 37°C for 2 h, the hybridoma cell supernatant was incubated with the primary antibody at 37°C for 1 h, after which the cells were washed thrice with PBST. HRP-conjugated goat anti-mouse IgG (Proteintech Group, China, 1:10,000 dilution) was added and incubated at 37°C for 1 h. After the wash step, the membranes were scanned on a near-infrared fluorescence scanning imaging system (Odyssey CLX, USA).

### 2.8 B-cell epitope identification via peptide mapping

To locate the recognition epitopes of the mAbs, a total of 28 overlapping peptides of the VP2 protein were manufactured by the Suzhou Epitope (Suzhou, China). There were 20 amino acids in length that overlapped each other by 10 amino acids, except for the final peptide, which was 14 amino acids in length. The short peptides were precoated (100 ng/well) in 96-well microplates, and the hybridoma cell supernatants were added as primary antibodies to test the binding activity via indirect ELISA as described above. Upon the identification of bound peptides, the peptides were further truncated into a series of peptides, which were subsequently cloned and inserted into the pCAGGS vector and expressed as HA-peptide-fused proteins in the eukaryotic expression system. Recombinant truncated proteins were expressed in HEK-293T cells and analyzed by Western blotting.

### 2.9 Alanine scanning

To determine which residues are crucial for mAb binding, a series of alanine mutations were generated in the indicated HA-tag fusion epitopes. The amino acids in the epitope were substituted with alanine. Therefore, 14 alanine mutants of the two epitopes were constructed. The point mutant proteins were expressed in HEK-293T cells, and the cell lysates were collected at 24 h post transfection and analyzed via Western blotting.

### 2.10 Generation of recombinant viruses

To further identify the amino acids that are critical for mAb 1B8 binding in virions, the infectious clone pSVA16 was used as a template for site-directed mutagenesis, and seven recombinant plasmids were constructed (pSVA-S177R, pSVA-L178A, pSVA-G179A, pSVA-T180A, pSVA-Y181A, pSVA-Y182A, and pSVA-R183A). BHK-21 cells seeded in 6-well plates were transfected with plasmids harboring pSVA16 or SVA mutants to rescue the recombinant viruses. Transfection was conducted using Lipofectamine^TM^ 3000 reagent following the manufacturer's instructions (Life Technologies, NY, USA). The cytopathic effect (CPE) was monitored daily after infection, and mutant viruses were harvested when significant CPE was observed. The recovered viruses were passaged ten times in BHK-21 cells, and the stability of the introduced mutations was confirmed by sequencing the VP2 coding region. The binding activity of the mAbs to the recombinant viruses was analyzed by IFA and Western blotting. The SVA VP1 mAb was preserved in our laboratory.

### 2.11 Biological information analysis

A total of thirty representative SVA strains were collected from the NCBI database for sequence comparison. The epitopes identified by the 1B8 and 2C7 mAbs were compared using BioEdit software to clarify the conserved characteristics of the epitopes among the different SVA strains. Subsequently, the SVA-VP2 amino acid sequence was submitted to the SWISS online system to predict its structural models through template searching and modeling. PyMOL software was used to construct the SVA VP2 three-dimensional (3D) structural model.

## 3 Results

### 3.1 Purification of the SVA recombinant VP2 protein and SVA virions

**Figure 1:**
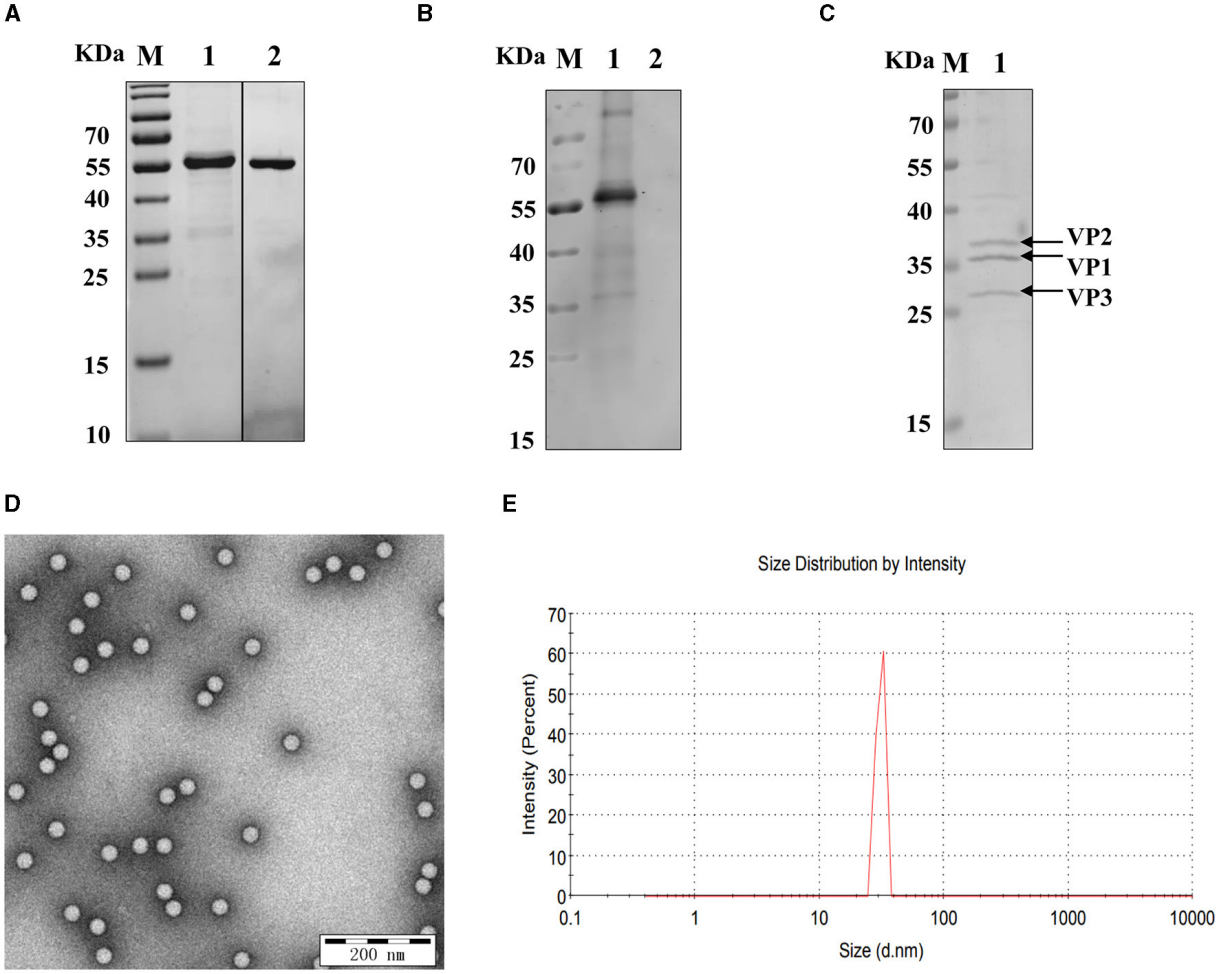
Identification of recombinant VP2 protein and SVA virions. **(A)** The purity of the SVA VP2 protein was analyzed by SDS–PAGE and visualized by Coomassie blue staining. M: protein marker; 1: reVP2 purified by Ni^2+^ column affinity chromatography; 2: reVP2 purified by size-exclusion chromatography (SEC). **(B)** Western blot analysis of the reactivity of the reVP2 protein with SVA-positive serum. 1: reVP2 protein; 2: pET-28a-SUMO protein. The purification of SVA virions was analyzed using SDS–PAGE and TEM. **(C)** SDS–PAGE. 1: Purification of SVA. **(D)** TEM image of SVA virions (×33,000), bar: 200 nm. **(E)** SVA particle size analysis via a nanoparticle size analyzer.

The SUMO-fusion recombinant VP2 protein (reVP2) cloned and inserted into the pET-28a (+) vector was successfully expressed in *E. coli* BL21 (DE3). The reVP2 protein was purified by Ni^2+^ column affinity chromatography and size-exclusion column (SEC) electrophoresis and analyzed via SDS–PAGE (Figure 1A). The molecular weight of the reVP2 protein was 55 kDa, which was consistent with the predicted recombinant protein size, and the purified reVP2 protein was detected as a single band by SDS–PAGE at a final concentration of 0.5 mg/mL. In addition, Western blot analysis was performed using SVA-positive pig sera, and the results confirmed that reVP2 could be specifically recognized by SVA antiserum, which indicated that the reVP2 protein had good reactivity with the antibody (Figure 1B). To screen for monoclonal antibodies that recognize the virus well, SVA virions were purified via CsCl gradients by ultrahigh-speed centrifugation, and the purified SVA virions were confirmed via SDS–PAGE (Figure 1C) and TEM (Figure 1D). Three specific bands were observed for high-purity virus particles by SDS–PAGE, and the protein sizes were ~39, 37, and 34 kDa, which are consistent with the known sizes of the SVA VP2, VP1, and VP3 proteins. In addition, the purified SVA was analyzed by a nanoparticle size analyzer, and the virus particles were ~ 30 nm in diameter (Figure 1E).

### 3.2 Characterization of mAbs against the VP2 protein

**Figure 2:**
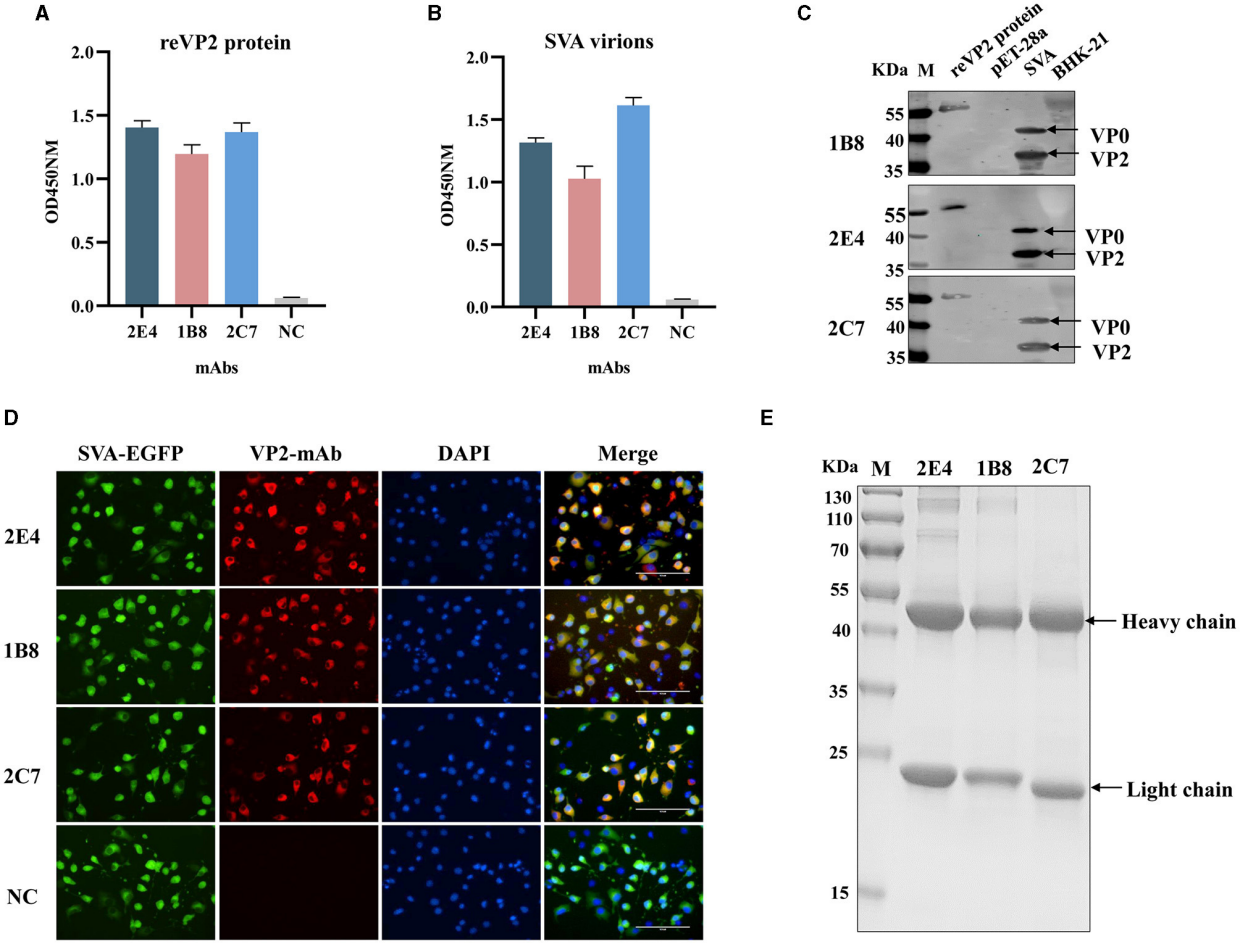
Functional analysis of monoclonal antibodies. The 2E4, 1B8, and 2C7 mAbs were used as primary antibodies in an indirect ELISA to test the reactivity with **(A)** the reVP2 protein and **(B)** the virus. NC: Negative control and SP2/0 cell supernatants were used as primary antibodies. All the experiments were repeated three times, and the error bars represent the standard deviation (SD). **(C)** The cell lysates of SVA-infected BHK-21 cells and reVP2 protein were collected for Western blotting, and the cell lysates of mock-infected BHK-21 cells and *E. coli* BL21 were used as control groups. **(D)** BHK-21 cells were infected with SVA-EGFP for 12 h. Then, the cells were fixed and incubated with the mAbs 2E4, 1B8, and 2C7 or with the supernatants of the SP2/0 cells as primary antibodies. NC: the supernatants of the SP2/0 cells. **(E)** SDS-PAGE analysis of purification of ascitic fluid produced by mAbs 2E4, 1B8, and 2C7. The two bands indicate heavy chain (55 KDa) and light chain (23 KDa) respectively.

Three monoclonal antibodies against VP2 were obtained, namely, 2E4, 1B8, and 2C7. All the mAbs exhibited similar binding activity to the reVP2 protein (Figure 2A), while 2C7 exhibited increased binding activity to SVA particles (Figure 2B). In addition, the heavy-chain and light-chain isotypes of all the mAbs were IgG1 and kappa (Table 1). Western blot analysis confirmed that the three mAbs strongly reacted with the denatured reVP2 protein and SVA-infected BHK-21 cell lysates (Figure 2C). Because precursor VP0 was cleaved into VP2 and VP4 proteins to assemble into full capsids, the VP0 protein is also recognized by antibodies in cell lysates. These results suggested that these mAbs recognized mainly linear epitopes on the capsid protein VP2 of SVA. Furthermore, to confirm the specificity of the three mAbs, IFA was carried out. Briefly, EGFP-SVA-infected BHK-21 cells were incubated with the primary antibodies 2E4, 1B8, and 2C7. The results demonstrated that SVA-infected cells (green) were recognized by 2E4, 1B8, and 2C7 (red), and specific red fluorescence signals were localized to cells expressing green fluorescence, but no red fluorescence was detected in the negative control (Figure 2D). Furthermore, the ascites fluid was purified by Protein G affinity column and identified by SDS-PAGE. The results showed heavy and light chains were detected in the purified ascitic fluid of all three antibodies, and the heavy chain was 55 kDa and the light chain was about 23 kDa (Figure 2E).

**Table 1:** The results of monoclonal antibody isotype ELISA.

**OD450**	**IgG 1**	**IgG 2a**	**IgG 2b**	**IgG 3**	**IgM**	**IgA**	**Kappa**	**Lambda**
2E4	1.784	0.133	0.073	0.105	0.099	0.227	0.966	0.068
1B8	1.574	0.084	0.081	0.083	0.095	0.089	0.892	0.058
2C7	2.084	0.092	0.083	0.108	0.103	0.095	1.089	0.066

### 3.3 Epitope mapping recognized by the mAbs

**Figure 3:**
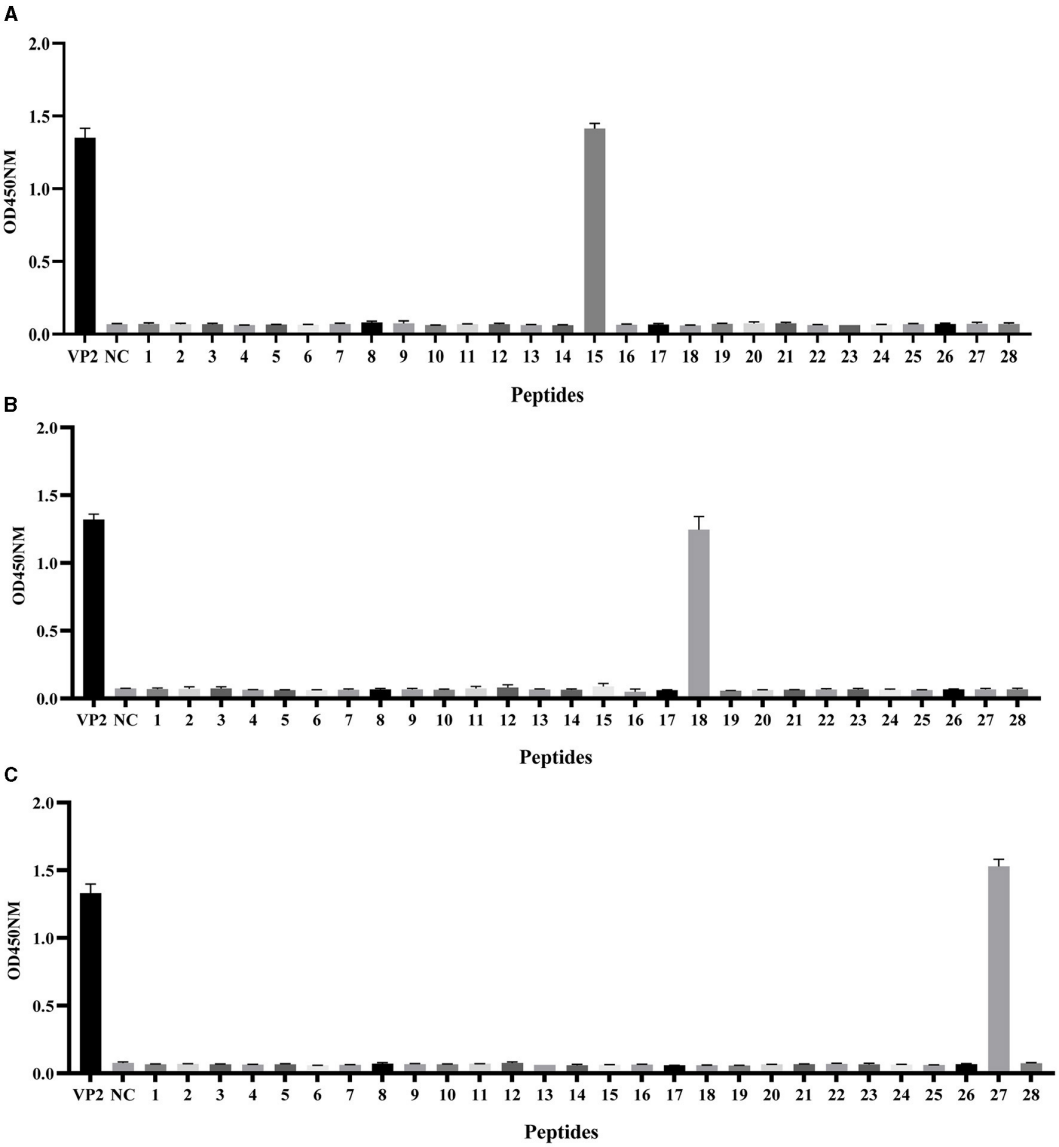
Identification of VP2 protein linear epitopes via PepScan. Reaction of the **(A)** mAb 2E4, **(B)** mAb 1B8, and **(C)** mAb 2C7 with 28 synthetic VP2 peptides. All the experiments were repeated three times, and the error bars represent the standard deviation (SD). The NC was an unrelated synthetic peptide.

To preliminarily screen the linear epitopes recognized by the three mAbs, 28 synthetic peptides (Table 2), each of which is an 20-mer with 10 overlapping residues at the ends that span the entire VP2 region, was used to screen the binding activity of the mAbs. The results demonstrated that the three mAbs recognized three different epitope peptides of VP2. 2E4 strongly bound to peptide 15 (^141^LDVRPDGKAKSLEELNEEQW^160^) (Figure 3A). The mAb 1B8 exhibited increased binding activity to peptide 18 (^171^KNMPFQSLGTYYRPPNWTWG^190^) (Figure 3B). In addition, 2C7 strongly responded to peptide 27 (^261^SVRPTSPYFNGLRNRFTTGT^280^) (Figure 3C).

**Table 2:** List of synthetic peptides of VP2.

**Peptide name**	**Position**	**Sequences**
VP2-1	1–20	DHNTEEMENSADRVITQTAG
VP2-2	11–30	ADRVITQTAGNTAINTQSSL
VP2-3	21–40	NTAINTTQSSLGVLCAYVEDP
VP2-4	31–50	GVLCAYVEDPTKSDPPSSST
VP2-5	41–60	TKSDPPSSSTDQPTTTFTAI
VP2-6	51–70	DQPTTTFTAIDRWYTGRLNS
VP2-7	61–80	DRWYTGRLNSWTKAVKTFSF
VP2-8	71–90	WTKAVKTFSFQAVPLPGAFL
VP2-9	81–100	QAVPLPGAFLSRQGGLNGGA
VP2-10	91–110	SRQGGLNGGAFTATLHRHFL
VP2-11	101–120	FTATLHRHFLMKCGWQVQVQ
VP2-12	111–130	MKCGWQVQVQCNLTQFHQGA
VP2-13	121–140	CNLTQFHQGALLVAMVPETT
VP2-14	131–150	LLVAMVPETTLDVRPDGKAK
VP2-15	141–160	LDVRPDGKAKSLEELNEEQW
VP2-16	151–170	SLEELNEEQWVEMSDDYRTG
VP2-17	161–180	VEMSDDYRTGKNMPFQSLGT
VP2-18	171–190	KNMPFQSLGTYYRPPNWTWG
VP2-19	181–200	YYRPPNWTWGPNFINPYQVT
VP2-20	191–210	PNFINPYQVTVFPHQILNAR
VP2-21	201–220	VFPHQILNARTSTSVDISVP
VP2-22	211–230	TSTSVDISVPYIGETPTQSS
VP2-23	221–240	YIGETPTQSSETQNSWTLLV
VP2-24	231–250	ETQNSWTLLVMVLVPLVPLDYKE
VP2-25	241–260	MVLVPLVPLDYKEGATTDPEITF
VP2-26	251–270	GATTDPEITFSVRPTSPYFN
VP2-27	261–280	SVRPTSPYFNGLRNRFTTGT
VP2-28	271–290	GLRNRFTTGTDEEQ

The epitope located at peptide 15 ^141^LDVRPDGKAKSLEELNEEQW^160^ has been reported to be a crucial neutralizing epitope (Wen et al., 2022), but mAb 2E4 showed no neutralizing activity against SVA (data not shown). Although, mAb 1B8 and 2C7 also had no neutralizing activity, the epitopes contained in peptides 18 and 27 recognized by these two mAbs have not yet been identified. Hence, we focused on precisely identifying the corresponding core epitopes of mAbs 1B8 and 2C7. The complete SVA VP2 gene was cloned and inserted into a eukaryotic expression vector with an HA tag as a template and a positive control for truncated peptides. A series of truncated peptides were transfected into HEK-293T cells, and the HA-fusion truncated peptides were identified via Western blotting.

**Figure 4:**
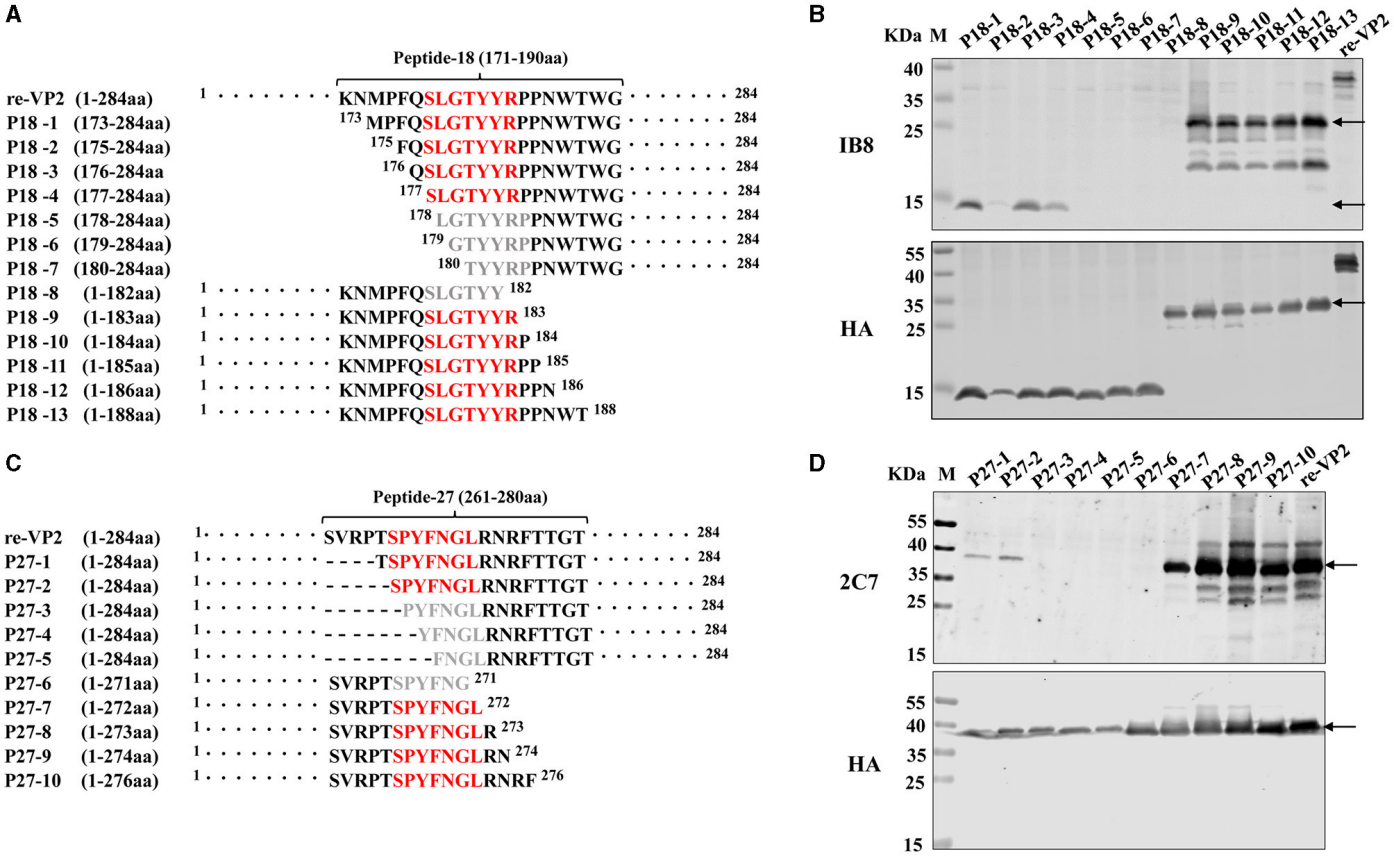
Mapping of the B-cell epitope by truncating short peptides. **(A)** Schematic diagram of peptide 18 (^171^KNMPFQSLGTYYRPPNWTWG^190^) used for epitope mapping, “·” represents the abbreviated amino acid. **(B)** HEK-293T cells were transfected with plasmids encoding thirteen truncated fragments for 24 h. The cell lysates were analyzed by Western blotting using an HA antibody and mAb 1B8. **(C)** Schematic diagram of peptide 27 (^261^SVRPTSPYFNGLRNRFTTGT^280^) used for epitope mapping. “·” represents the abbreviated amino acid and “-” represents the deleted amino acid. **(D)** HEK-293T cells were transfected with plasmids encoding five truncated fragments for 24 h. The cell lysates were analyzed by Western blotting using an HA antibody and the mAb 2C7.

To precisely identify the core sequence recognized by the mAb 1B8, the VP2 protein was sequentially truncated at the position of peptide 18 from the N-terminus and C-terminus. The length and position of these truncated fragments in VP2 were shown in Figure 4A. A total of 13 truncated fragments based on peptide 18 were used to investigate the binding activity to 1B8. For peptide 18, N-truncated fragments bind mAb 1B8 effectively until residue Ser^177^ is removed (P18-5), and C-truncated fragments of peptide 18 bind 1B8 strongly until residue Arg^183^ is removed (P18-8) (Figure 4B). These results indicated that the peptide ^177^SLGTYYR^183^ was the minimal residue for binding to mAb 1B8.

To map the core sequence recognized by the mAb 2C7, 10 truncated fragments were used to determine the binding activity. Because the peptide 27 was located at the VP2 protein's C-terminus, the N-terminus of peptide 27 was subsequently truncated from full-length VP2 protein (Figure 4C). Deletion of Ser^266^ led to a decrease in the immunoreactivity of the N-truncated fragment P27-3 compared with that of P27-2 (Figure 4D). Moreover, the mAb 2C7 effectively recognized the C-truncated fragments until Leu^272^ was removed (P27-6). The results indicated that the minimal residue recognized by mAb 2C7 was ^266^SPYFNGL^272^.

### 3.4 Critical residues of the linear epitopes responsible for mAb binding

**Figure 5:**
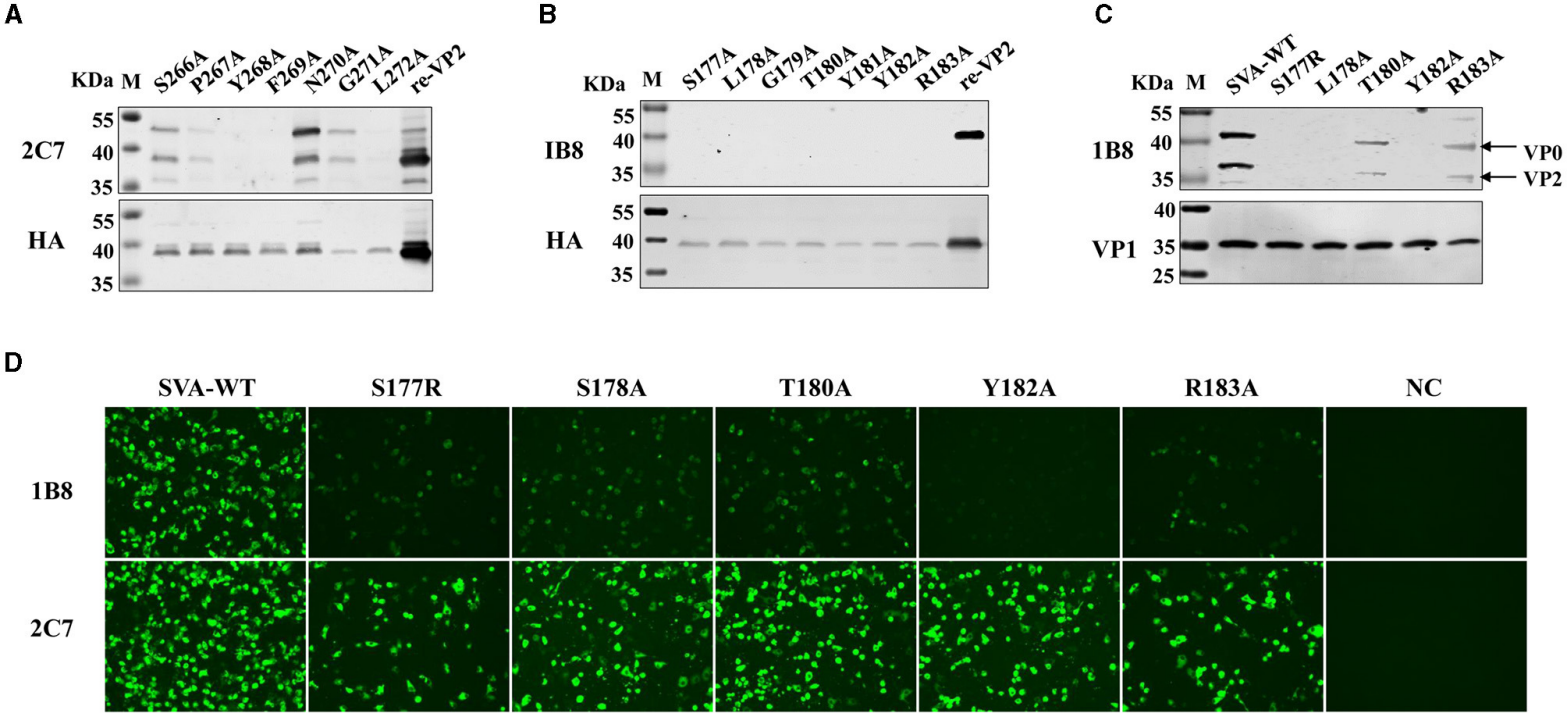
Identification of the pivotal points in the epitopes. Single alanine substitution scanning of the HA-fused VP2 protein was carried out, and the proteins were identified via Western blotting. **(A)** Binding ability of the mAb 2C7 to VP2 mutants. **(B)** Binding capacity of the mAb 1B8 to VP2 mutants. **(C, D)** BHK-21 cells were infected with SVA-WT or the SVA mutant virus at an MOI of 0.1 for 24 h, and the reactivity of the mAb 1B8 to the single-site mutation SVA was analyzed by **(C)** Western blotting and **(D)** Immunofluorescence analysis; NC: mock-infected cells.

Next, the critical residues in these two novel epitopes responsible for mAb binding were analyzed. Single alanine substitution scanning of the HA-fused VP2 protein was carried out, and the protein was identified via Western blotting. The mAb 2C7-recognized epitope (^266^SPYFNGL^272^) was subjected to continuous alanine mutation to construct alanine mutants. The results confirmed that the protein contains a discontinuous key amino acid. The Ser^266^, Asn^270^, and Gly^271^ mutants reacted strongly with the mAb 2C7, and the Pro^267^ mutant reacted relatively weakly with 2C7 (Figure 5A). However, the mutants Tyr^268^, Phe^269^, and Leu^272^ were unable to bind to the mAb 2C7. These results indicate that the residues Tyr^268^, Phe^269^, and Leu^272^ are critical amino acids for recognition of the VP2 protein by mAb 2C7.

However, the ^177^SLGTYYR^183^ epitope contains a continuous key amino acid, and the residues from Ser^177^ to Arg^183^ with alanine substitution strongly affect the reactivity of the VP2 epitope recognized by 1B8 but not by the anti-HA antibody (Figure 5B), indicating that all the residues are necessary for the binding of VP2 to mAb 1B8. These results indicate that ^177^SLGTYYR^183^ may be a potential epitope for the development of a marker vaccine. Therefore, the reactivity of point mutant viruses located at the VP2 ^177^SLGTYYR^183^ epitope with the mAb 1B8 was further analyzed. We successfully rescued five mutant viruses (S177R, L178A, T180A, Y182A, and R183A), but substituting arginine or alanine at residues 179 and 181 failed to afford the mutant virus, which indicates that residues Gly^179^ and Arg^181^ of VP2 are crucial for the replication of SVA. By Western blotting, we found that mAb 1B8 strongly binds to SVA-WT and much weakly binds to the T180A and R183A mutants. No binding of the mAb 1B8 to the S177R, L178A, or Y182A mutant was detected (Figure 5C). However, the expression of the VP1 protein in both SVA-WT and SVA-mutant-infected cells was detected. In comparison, the mAb 2C7 strongly reacted to the mutant viruses and SVA-WT, while the mAb 1B8 exhibited notably lower reactivity to the mutant viruses than did SVA-WT. The Y182A mutation in particular completely abrogated the ability of the mAb 1B8 to bind to the virus (Figure 5D), which suggested that the Tyr^182^ residue is involved in the interaction between SVA and the mAb 1B8. Our results suggest that the combination of the Ser^177^, Leu^178^, and Tyr^182^ residues may be an ideal mutation site for the generation of epitope-based SVA marker vaccines.

### 3.5 The identified epitopes are highly conserved in SVA VP2 proteins

**Figure 6:**
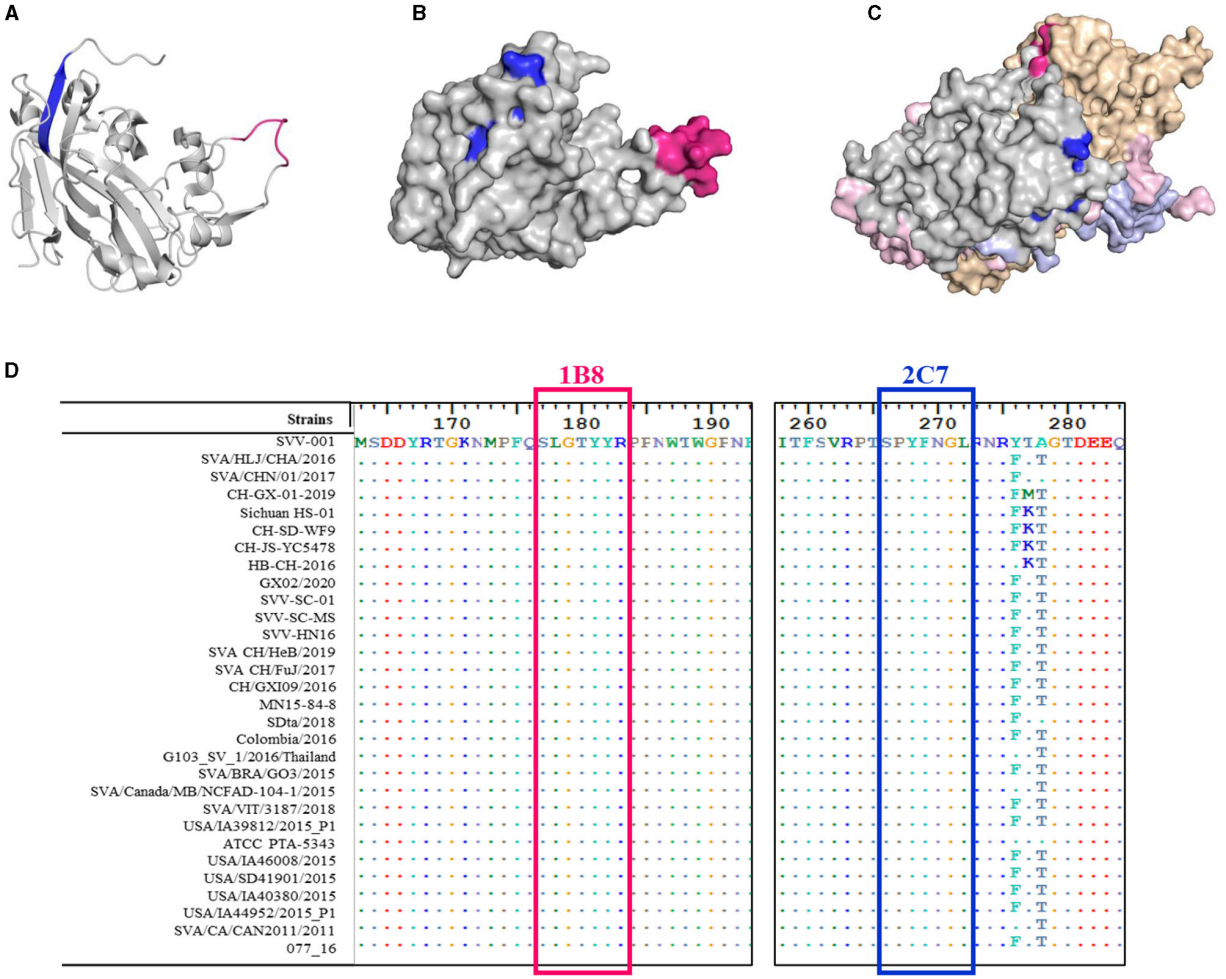
Spatial distribution and conservation of the identified epitopes. 3D model of the SVA VP2 protein. The epitope ^177^SLGTYYR^183^ is marked in red, and the epitope ^266^SPYFNGL^272^ is marked in blue and drawn as **(A)** a secondary structural analysis of the two epitopes on the VP2 monomer skeleton and **(B)** stereo-structures of the two epitopes in the VP2 protein monomer (gray). **(C)** Stereo-structures of the two epitopes in the capsid protomer with VP1 (light brown), VP3 (pink), and VP4 (purple). **(D)** Amino acid alignment of the VP2 protein was performed for different SVA strains. Only the different residues are shown. The red box indicates the epitope recognized by mAb 1B8, and the blue box indicates the epitope recognized by mAb 2C7.

In addition to the amino acid sequence, the antigenicity of the epitope is related to the spatial structure of the epitope. The structural homology model of the VP2 protein was constructed by SWISS, and the one with the highest confidence (PBD:3CJI) was selected for subsequent analysis. The spatial structure was displayed with PyMoL software. The epitope ^177^SLGTYYR^183^ (marked in pink) formed a random coil (Figure 6A) that was fully exposed on the surface of the VP2 protein (Figure 6B). In addition, this epitope is located at the interface area of the ANTXR1-SAV binding site (Jayawardena et al., 2018) and is exposed on the surface of the capsid protomer with VP1, VP2, VP3, and VP4 (Figure 6C). In contrast to the epitope at ^177^SLGTYYR^183^, the epitope at ^266^SPYFNGL^272^ (marked in blue) formed part of a β-sheet (Figure 6A). The surface of the VP2 protein was partially exposed, and the middle region was obscured by the amino acid ^52^QPTTT^56^ (Figure 6B); this feature determines whether the position of the crucial amino acid that binds to the antibody is discontinuous.

To determine the conservation of these two epitopes, the VP2 gene of SVA from China and other countries was collected from GenBank, and the conservation of these epitopes was analyzed via BioEdit software. The epitopes ^177^SLGTYYR^183^ and ^266^SPYFNGL^272^ were highly conserved between SVA strains, and they exhibited 100% sequence similarity (Figure 6D). These results suggest that these residues are important for maintaining epitope structure or function.

## 4 Discussion

SVA is prevalent in many countries, and SVA-associated vesicular diseases have caused economic losses to the pig industry. In this study, two novel linear epitopes of VP2 were precisely identified by mAbs. Importantly, we confirmed that the epitope ^177^SLGTYYR^183^ is located at the VP2 “puff” loop region and contains key residues involved in receptor binding. In addition, the single mutation Y182A abolishes the interaction between the mutant virus and the mAb 1B8. Therefore, our findings could lead to the development of an epitope-based SVA marker vaccine to distinguish infected pigs from vaccinated pigs.

Monoclonal antibodies with high specificity and sensitivity are widely used in the diagnosis and treatment of diseases, such as Alzheimer's disease (AD), have produced encouraging cognitive and clinical results (Qiao et al., 2024). Besides, engineering modifications of mAbs significantly improve their therapeutic properties, which has emerged as a promising strategy to enhance tumor targeting specificity and immune cell interactions (Mathieu et al., 2024). In addition, mAbs are widely applied for the establishment of immunological detection methods for infectious or non-infectious diseases. At the same time, mAbs as an important tool have been used in identifying antigenic epitopes. The gold standard for epitope definition is X-ray analyses of crystals of antigen-antibody complexes, however, this method requires a high degree of sophistication and expertise. Most other methods rely on monitoring the binding of the antibody to antigen fragments. The epitope identification will benefit to the study of pathogen-host interaction mechanism and the structure and function of target proteins.

To date, several antigenic epitopes of SVA VP2 have been identified through bioinformatics prediction and synthetic polypeptide therapy using mAbs or porcine serum (Fan et al., 2020; Ru et al., 2023; Zhang et al., 2023). The ^141^LDVRPDGKAKSLEELNEEQW^160^ region is a dominant epitope region with relatively abundant epitopes in the VP2 protein (Fan et al., 2020; Wen et al., 2022; Ru et al., 2023; Zhang et al., 2023). In addition, Wen et al. obtained five neutralizing mAbs by immunizing mice with ultra-purified SVA, and the linear epitope ^153^QELNEE^158^ recognized them. The amino acid residue Glu^157^ is the pivotal site for neutralizing SVA (Wen et al., 2022). However, Fan et al. (2020), reported that two mAbs generated by recombinant SVA VP2 protein immunization showed high affinity to peptide ^149^AKSLQELNE^157^, but did not have neutralizing activity. Similarly, we identified mAb 2E4, which strongly binds to ^141^LDVRPDGKAKSLEELNEEQW^160^, showed no neutralizing effects on SVA either. All these suggest that the antigen with conformational epitopes is more likely to induce the production of neutralizing mAb. Nevertheless, the epitope localized in ^141^LDVRPDGKAKSLEELNEEQW^160^ is an important immunodominant epitope and provides an important detection target for epidemiological investigations.

Importantly, the protruding part of the VP2 “puff” loop responsible for ANTXR1 binding is highly conserved. Based on the structure of the SVA-ANTXR1 complex, ANTXR1 interacts with three major capsid proteins and is centered on the “puff” loop of VP2 (Miles et al., 2017; Cao et al., 2018). Jayawardena et al. suggested that residues 178–186 contribute to the bulk of receptor interactions, and residues Leu^178^, Thr^180^, Tyr^182^ and Pro^184^ on the “puff” loop of VP2 form van der Waals interactions or aromatic interactions with residues on the α4 helix of ANTXR1 (Jayawardena et al., 2018). The ANTXR1 binding site can be recognized by neutralizing mAbs, which suggests that the ANTXR1 binding site on SVA capsids may partially overlap with the epitope of neutralizing antibodies. We identified a linear epitope (^177^SLGTYYR^183^) is located at VP2 receptor binding site, and this is the first minimal epitope on the VP2 “puff” loop identified using the mAb 1B8. However, the mAb 1B8 has no neutralizing effect on SVA. Recently, Zhao et al. (2023), confirmed that mAb 2G6 exhibits strong neutralizing activity to SVA, which recognized conformational epitope, and the mAb heavy chains bind to VP2 and knob loop of VP3, respectively. It has been suggested that most conformational epitopes are composed of several linear epitopes (Liu et al., 2017; Ferdous et al., 2019). Therefore, ^177^SLGTYYR^183^ may constitute a segment with a conformational epitope that induces neutralizing antibody production. Investigating conformational epitopes and neutralizing antibody production in the “puff” loop of VP2 would be highly important for future work. In particular, identifying SVA mutants capable of evading neutralizing effects may benefit oncolytic virotherapy in humans (Zhao et al., 2023).

Furthermore, the C-terminal region of VP2 (^271^GLRNRFTTGTDEEQ^284^) was identified as a diagnostic target for testing SVA antibodies in pigs (Ma et al., 2022). In the present study, the minimal epitope ^266^SPYFNGL^272^ of the C-terminus of VP2 was identified accurately by the mAb 2C7, and two amino acids (Gly^271^ and Leu^272^) were found overlapping with previous study conducted by Ma et al. Recently, the epitope ^267^PYFNGLRNRFTTGT^280^ was predicted and identified by bioinformatics-based computational prediction and the Pepscan approach (Ru et al., 2023). However, we found that residue Ser^266^ is important for maintaining the binding of ^266^SPYFNGL^272^ to mAb 2C7. In addition, ^266^SPYFNGL^272^ was shown to be partially exposed on the VP2 protein, and the key amino acids analysis revealed that the immunodominant residues are associated with the immunogenicity and reactivity of the epitope. Our results suggest that the epitope ^266^SPYFNGL^272^ is an important and conserved B-cell immunodominant epitope.

In summary, two novel epitopes of SVA VP2, ^177^SLGTYYR^183^ and ^266^SPYFNGL^272^, were identified using mAbs. Importantly, we found that the epitope ^177^SLGTYYR^183^ completely exposed on the surface of the VP2 protein, and confirmed that the VP2 “puff” loop region harbors a BCE containing key amino acid residues involved in receptor binding. These results will help to elucidate the antigenic characteristics of VP2 and promote the development of diagnostic tools and DIVA vaccines for SVA.

## Data availability statement

The datasets presented in this study can be found in online repositories. The names of the repository/repositories and accession number (s) can be found below: https://www.ncbi.nlm.nih.gov/genbank/, DQ641257.1; KY419132.1; MG765550.1; MT457474.1; MH588717.1; MN882360.1; MN882361.1; KX377924.1; MW117126.1; MH716015.1; MN700930.1; MF893200.1; MZ375462.1; MH490944.1; KY038016.1; KU359211.1; MN433300; KX857728.1; KY368743.1; KR063109.1; KY486156.1; MH704432.1; KU954087.1; KU954086.1; KT757282.1; KT757281.1; KT757280.1; KU954090.1; MT360257.1; MF615504.1.

## Ethics statement

The animal study was approved by Animal Care and Ethics Committees of Harbin Veterinary Research Institute, Chinese Academy of Agricultural Sciences. The study was conducted in accordance with the local legislation and institutional requirements.

## Author contributions

HZ: Data curation, Formal analysis, Validation, Writing – original draft. MS: Data curation, Validation, Writing – review & editing. SS: Data curation, Formal analysis, Validation, Writing – review & editing. LM: Data curation, Writing – review & editing. WY: Funding acquisition, Validation, Writing – review & editing. LY: Data curation, Methodology, Writing – review & editing. XS: Formal analysis, Writing – review & editing. XL: Data curation, Formal analysis, Writing – review & editing. HW: Formal analysis, Validation, Writing – review & editing. HM: Formal analysis, Project administration, Writing – review & editing. XC: Formal analysis, Validation, Writing – review & editing. Y-DT: Project administration, Supervision, Writing – original draft. TA: Funding acquisition, Project administration, Supervision, Writing – review & editing. FM: Funding acquisition, Supervision, Writing – original draft.
